# Effect of Controlling Thiophene Rings on D-A Polymer Photocatalysts Accessed via Direct Arylation for Hydrogen Production

**DOI:** 10.3390/molecules28114507

**Published:** 2023-06-01

**Authors:** Dongnai Ye, Lei Liu, Qimin Peng, Jiabin Qiu, Hao Gong, Aiguo Zhong, Shiyong Liu

**Affiliations:** 1 Jiangxi Provincial Key Laboratory of Functional Molecular Materials Chemistry, College of Materials, Metallurgical and Chemistry, Jiangxi University of Science and Technology, Ganzhou 341000, China; yedongnai2014@gnnu.edu.cn (D.Y.); qm2474572831@163.com (Q.P.); 6720210821@mail.jxust.edu.cn (H.G.); 2 School of Chemistry and Chemical Engineering, Gannan Normal University, Ganzhou 341000, China; 3 Department of Pharmacy & Chemistry, Taizhou University, Taizhou 318000, China; zhongaiguo@tzc.edu.cn

**Keywords:** direct C–H arylation polymerization, donor–acceptor linear conjugated polymers, thiophene derivatives, photocatalysis, visible-light-driven hydrogen evolution

## Abstract

Conjugated polymer photocatalysts for hydrogen production have the advantages of an adjustable structure, strong response in the visible light region, adjustable energy levels, and easy functionalization. Using an atom- and step-economic direct C–H arylation method, dibromocyanostilbene was polymerized with thiophene, dithiophene, terthiophene, and fused thienothiophene and dithienothiophene, respectively, to produce donor–acceptor (D-A)-type linear conjugated polymers containing different thiophene derivatives with different conjugation lengths. Among them, the D-A polymer photocatalyst constructed from dithienothiophene could significantly broaden the spectral response, with a hydrogen evolution rate up to 12.15 mmol h^−1^ g^−1^. The results showed that the increase in the number of fused rings on thiophene building blocks was beneficial to the photocatalytic hydrogen production of cyanostyrylphene-based linear polymers. For the unfused dithiophene and terthiophene, the increase in the number of thiophene rings enabled more rotation freedom between the thiophene rings and reduced the intrinsic charge mobility, resulting in lower hydrogen production performance accordingly. This study provides a suitable process for the design of electron donors for D-A polymer photocatalysts.

## 1. Introduction

Photocatalytic water splitting is considered a promising technology to access clean hydrogen energy. Despite great efforts, the development of solar-driven photocatalysts with sufficient activity is still one of the most important goals pursued by scientists. Photocatalysts can be typically divided into two categories, i.e., inorganic and organic semiconductor photocatalysts. Compared with inorganic semiconductors, organic semiconductors, i.e., π-conjugated polymers, have some outstanding merits, including the large variety of synthesis methods, low pollution, and regulatable and controllable structures. Since poly (p-phenylene) [1] was reported as an active material, numerous organic semiconductors, such as linear conjugate polymers [2,3,4,5,6], conjugated microporous polymers [7,8,9,10,11], covalent organic frameworks [12,13,14,15], covalent triazine frameworks [16,17,18,19], metal–organic frameworks [20,21,22,23], and others [24,25,26], have been developed as photocatalysts for hydrogen evolution. Photocatalytic water splitting by using polymeric semiconductors to produce hydrogen has promising applications, but it is still in the fundamental stage at a laboratory scale. The exploration of appropriate building blocks remains a challenge for developing highly efficient polymeric photocatalysts.

Thiophene is one of the most popular electron-donating building blocks in organic semiconductors due to its Π56 planar structure and appropriate aromatic stabilization energy, which can enhance the electron density on conjugated chains and promote charge carrier transport [27]. Meanwhile, owing to its flexible, compact, and planar conformation, thiophene can be facilely adjusted by introducing different numbers of thienyl rings when forming π-conjugated chains. Additionally, thiophene derivatives can broaden light absorption [28], which will promote the light-harvesting ability of photocatalysts. In addition, fused thiophene (such as dithieno [3,2-b:2′,3′-d] thiophene) has a more rigid structure, extended π-conjugation system and light absorption, and higher electron transport capacity, which will facilitate photocatalytic hydrogen production (PHP) [29].

Diarylethenes are a type of important building block for polymer semiconductors, which have high charge mobility for organic electronic devices, e.g., poly(thienylenevinylenes) [30]. Diarylethylenes containing both aromatic and vinyl units can effectively extend the conjugation lengths of polymer systems. In addition, the presence of vinyl bonds in the main aromatic polymeric chain can increase the degree of coplanarity of the main polymer chain, as vinyl bonds can reduce spatial hindrance on continuous aromatic rings. Our studies have revealed that 2-styrylthiophene-based linear conjugated polymers (CPs) with a –CN substituent have impressive PHP performance [31,32]. The dibromocyanostilbene containing electron-withdrawing cyano groups in the ethylene position, as diarylethene derivatives with D-A architecture, is expected to serve as an excellent skeleton for D-A CP-based efficient photocatalysts in PHP reactions. Meanwhile, dibromocyanostilbene moieties have the distinct advantage of facile synthesis via Knoevenagel condensation [31].

**Scheme 1:**
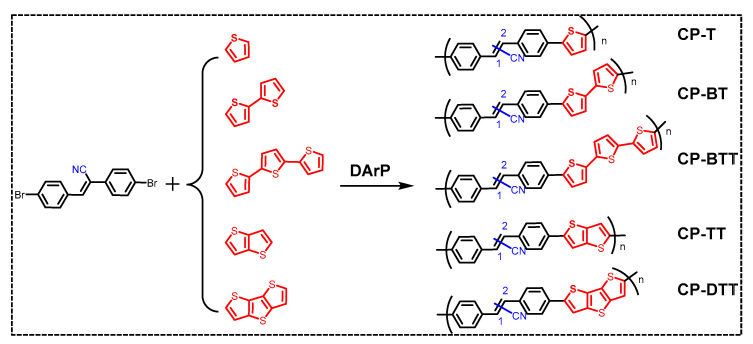
Synthesis routes of **CP-T**, **CP-BT**, **CP-BTT**, **CP-TT**, and **CP-DTT** via DArP.

Conjugated polymer photocatalysts can be synthesized by Suzuki coupling, Stille coupling, the Sonogashira coupling reaction, direct C–H arylation polymerization (DArP), etc. Among them, the DarP strategy has the advantages of simplified synthetic steps, higher atomic economy, and lower cost, which conforms well to the 12 principles of green chemistry for avoiding side effects and using toxic reagents [33,34,35]. In continuation of our previous efforts on diarylethene-based D-A CP photocatalysts [31,32], here, we try to disclose the effect of different thiophene-based building blocks on the PHP of D-A conjugated polymers. Five linear CPs (i.e., **CP-T**, **CP-BT**, **CP-BTT**, **CP-TT,** and **CP-DTT**) were facilely obtained from atom- and step-economic DArP of dibromocyanostilbene (**DBCS**) with thiophene (**T**), bithiophene (**BT**), 2,2′:5′,2″-terthiophene (**BTT**), thieno[3,2-b] thiophene (**TT**), and dithieno[3,2-b:2′,3′-d] thiophene (**DTT**), respectively (Scheme 1). The opto-electrochemical measurements and PHP test were systematically studied for these DArP-derived CPs with tunable thiophene rings in the structure–property–performance correlations, showing that **CP-DTT** possessing the highest PHP performance was achieved when water with a sacrificial electron donor (SED) and ascorbic acid (AA) were used as the proton source. The results obtained here will provide a suitable guideline for the design of thiophene-containing CP photocatalysts for PHP applications.

## 2. Results and Discussion

### 2.1. Synthesis and Characterization of the **CPs**

As shown in Scheme 1, **DBCS** bearing an electron-withdrawing –CN group was facilely synthesized via Knoevenagel condensation [36]. The structure of the **DBCS** was confirmed by NMR spectra (Figures S1 and S2). D-A linear conjugate polymers of **CP-T**, **CP-BT**, **CP-BTT**, **CP-TT**, and **CP-DTT** were obtained, respectively, by using thiophene (**T**), unfused **BT** and **BTT**, and fused **TT** and **DTT** as electron-donating building blocks to polymerize with electron-accepting **DBCS**.

**Figure 1:**
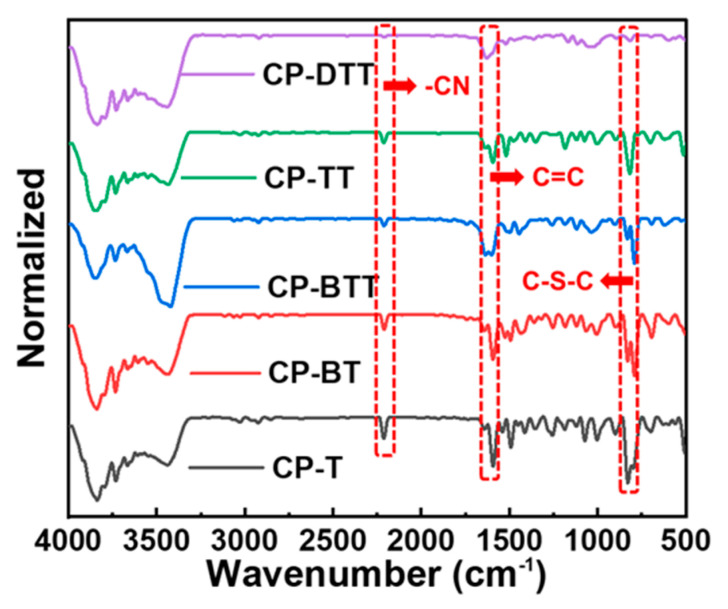
FT-IR spectra of CPs.

The chemical structures of all CPs were further verified by Fourier-transform infrared (FT-IR) spectra (Figure 1). All CPs exhibited peaks at a wavelength of ~816 cm^−1^, corresponding to the characteristic peak of the stretching vibration of C-S-C. The characteristic signal of the C=C double bonds stretching mode appeared at ~1635 cm^−1^, demonstrating the presence of vinylene linkers. Additionally, the signals at ~2215 cm^−1^ were assigned to the stretching vibration bands of the C≡N triple bond. The CPs displayed peaks at ~3420 cm^−1^, which suggested the successful incorporation of thiophene units at the α position into all CPs. To sum up, the FT-IR signals of all the above indicated that the target CPs were successfully synthesized via direct C–H arylation.

With the above structural and electronic features, thiophene derivatives can be used to finely control the optical band gap of the photocatalysts. Furthermore, the linear conjugate polymers possessed the merits of easy synthesis, structural simplicity, and atom- and step-economic and efficient D-A architecture. These polymers containing thiophene-based units with different conjugations (Scheme 1) are expected to be ideal models for structure–property–performance correlation studies via the PHP reaction.

**Figure 2:**
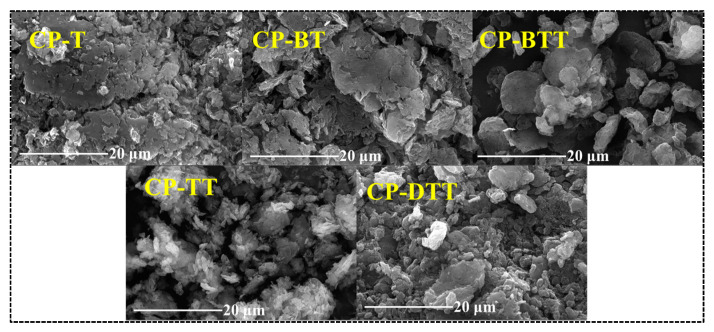
SEM images of CPs.

The morphologies of the powdery CPs were evaluated by scanning electron microscopy (SEM). Well-defined dimensions and aggregation are displayed for the CPs in Figure 2. Owing to the conjugation effect, unfused thiophene groups can easily adjust their conformation to remain coplanar with the benzene ring; thus, **CP-T**, **CP-BT**, and **CP-BTT** exhibited a lamella-like morphology. Normally, high crystallinity is conducive to obtaining efficient charge transport and separation of carriers for PHP, as organic polymers with high crystallinity can promote the separation and transport of photo- generated charge carriers due to the minimized formation of defects and charge traps. Otherwise, owing to the rigid structure, the fused **CP-TT** and **CP-DTT** exhibited nanoparticle morphology, which should have resulted in a higher HER. Furthermore, by involving the thiophene units in the conjugate backbone, the SEM morphologies of unfused **CP-T**, **CP-BT**, and **CP-BTT** were different, suggesting that the variation in the thiophene number had an influence on their morphologies.

### 2.2. Opto-Electronic Properties of the **CPs**

**Figure 3:**
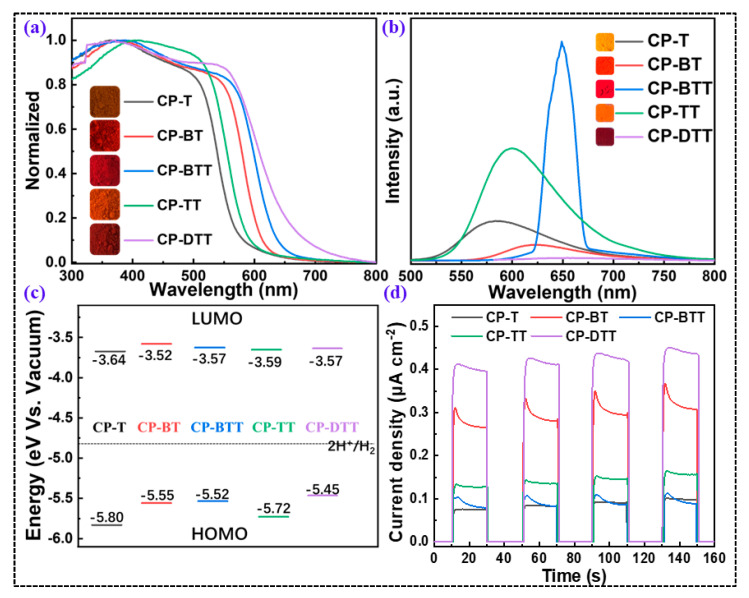
UV-vis DRS (**a**) and steady-state PL (**b**) spectra, FMO alignments (**c**), and TPR analysis (**d**) of CPs. (Insets in (**a**,**b**) show photos of the photocatalysts under ambient conditions and 365 nm irradiation, respectively.)

To shed light on the effect of different thiophene units on the opto-electronic properties, all the CPs were systematically characterized by different measurements, including UV–vis, steady-state photoluminescence (PL), the transient photocurrent response (TPR), and cyclic voltammetry (CV) (Figure 3 and Figure S3). A broader range of light absorption for a photocatalyst will be beneficial to photon capture and utilization [29]. Longer conjugated systems involving larger numbers of thiophene rings will lead to the enhanced delocalization of π-conjugation and result in a red shift of UV–vis absorption [27], which should be conducive to light harvesting by photocatalysts. The UV–vis diffuse reflectance spectroscopy of the CPs exhibited broad absorption between 300 and 600 nm. Compared with **CP-T**, the other CPs showed significant red-shifts. The range of light absorption for all CPs decreased in the order of **CP-DTT** > **CP-BTT** > **CP-BT** > **CP-TT** > **CP-T**. The varied planarity, electron-donation abilities, and conjugation lengths of **T**, **BT**, **BTT**, **TT**, and **DTT** endowed their corresponding parent CPs, i.e., **CP-T**, **CP-BT**, **CP-BTT**, **CP-TT**, and **CP-DTT**, with different rigidities, light absorptances, and electron separation capacity, and thus finely modulated their opto-electronic properties. As a result, **CP-DTT** exhibited the broadest absorption range, even with an extension to the near-infrared region, owing to the stronger electron-donation ability of **DTT** and the enhanced D-A interaction and interchain π-π* transition. The light absorption trends of the CPs were reflected by the evolution of colors, i.e., stronger absorbance toward longer wavelengths corresponded to deeper colors (insets in Figure 3a). **CP-DTT** exhibited the most red-shifted light absorption with the deepest color, which should be conducive to light harvesting. The optical bandgaps (*E_g_*) estimated by plotting the curves of (*αhν*)^1/2^ vs. *hν* (Figure S4) for **CP-T**, **CP-BT**, **CP-BTT**, **CP-TT**, and **CP-DTT** were 2.16, 2.03, 1.95, 2.13, and 1.88 eV, respectively, which meant that the introduction of unfused **BT** and **BTT** and the fused **TT** and **DTT** could finely modulate the *E_g_* of the photocatalyst. In theory, the minimum photon energy required to drive the fully decomposed water reaction is 1.23 eV, corresponding to photons with a wavelength of approximately 1000 nm. However, in reality, owing to the influence of semiconductor band bending and the presence of water decomposition overpotential, the requirement for semiconductor *E_g_* is greater than the theoretical value, generally believed to be greater than 1.8 eV. Therefore, the *E_g_* of **CP-T**, **CP-BT**, **CP-BTT**, **CP-TT**, and **CP-DTT** is suitable for PHP reactions.

PL spectroscopy was used to examine the photoinduced electron–hole (e^−^-h^+^) recombination behavior. Photoluminescence is a simple, but useful, index to check the photo-to-photon conversion. A weak photoluminescence intensity usually means lower radiative recombination of e^−^-h^+^ pairs, indicating charge separation and, thus, photocatalysis [4]. Figure 3b shows the PL spectra of the powdery **CP-T**, **CP-BT**, **CP-BTT**, **CP-TT,** and **CP-DTT** excited by 437, 445, 460, 477, and 562 nm wavelengths, respectively. The PL intensities of all CPs decreased in the order of **CP-BTT** > **CP-TT** > **CP-T** > **CP-BT** > **CP-DTT. CP-DTT** had the lowest PL intensity, meaning that **CP-DTT** can effectively promote intramolecular charge separation and minimize radiation recombination. On the contrary, **CP-BTT** displayed the strongest PL emission, indicating a high ratio of radiative charge recombination.

A photocatalytic reaction is initiated by photon capture to generate excited electron–hole pairs (i.e., excitons), followed by their separation into free charges, which eventually drive redox reactions. Typically, the driving force for the photocatalytic redox reaction is governed by the FMOs of CPs, i.e., the HOMO levels and the lowest unoccupied molecular orbital (LUMO). HOMO and LUMO, with electron-donating and electron-withdrawing characteristics, are responsible for oxidation and reduction reactions, respectively [29]. Here, the changes in the FMOs with the structural evolution from **CP-T**, **CP-BT**, **CP-BTT**, and **CP-TT** to **CP-DTT** involving different thiophene building blocks were investigated by CV measurements. The LUMO levels were calculated as −4.80 − (*E_red_* − *E_Fc/Fc+_*), while the HOMO levels were calculated as *E_LUMO_* − *E_g_*. As shown in Figure 3c, versus the vacuum hydrogen electrode, the LUMO levels of all CPs were greater than −4.8 eV, all of which had sufficient driving forces for proton reduction in thermodynamics. Compared with the single thiophene (**T**), the unfused thiophenes (**BT** and **BTT**) and the fused thiophenes (**TT** and **DTT**) had stronger electron donating ability and higher HOMO levels, as predicted by DFT calculation. As a result, the **CP-BTT** and **CP-DTT** exhibited higher HOMO levels compared with the other three CPs, i.e., **CP-T**, **CP-BT**, and **CP-TT**. The above results imply that, besides optical properties (Figure 3a), the electrochemical properties (i.e., FMOs) can also be finely tuned by the introduction of different thiophene derivatives.

To check the photo-to-current efficiencies of the CPs, TPR measurements were carried out under visible light irradiation under 1.5 V and Ag/AgCl conditions through the alternating switch method. As shown in Figure 3d, the photo-to-current intensity of the five CPs followed the order of **CP-DTT** > **CP-BT** > **CP-BTT** > **CP-TT** > **CP-T**. Among them, **CP-DTT** (0.46 µA) and **CP-BT** (0.38 µA) showed higher and more stable photocurrents compared with the other three CPs. The highest TPR response of **CP-DTT** can be ascribed to the strongest electron-donating ability of **DTT**, which was conducive to yielding the highest intermolecular charge transport.

**Figure 4:**
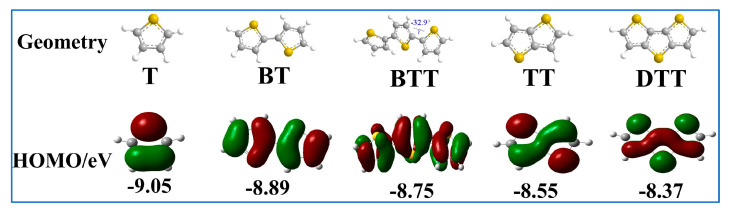
Optimized geometries and FMOs of the CPs predicted by DFT calculation.

To gain deeper insight into the structure–property correlation, we calculated the optimized geometries and the frontier molecular orbitals (FMOs) of the **T**, **BT**, **BTT**, **TT**, and **DTT** building blocks by the density functional theory (DFT) to reveal their effects on the opto-electronic properties of the polymers **CP-T**, **CP-BT**, **CP-BTT**, **CP-TT,** and **CP-DTT**. The DFT prediction revealed that **TT** and **DTT** possessed entirely planar geometries, while **BT** had rotating σ-bonds between the thiophene rings, and **BTT** had a twisted geometry with a dihedral angle of 32.9° (Figure 4). The highest occupied molecular orbital (HOMO) levels of the thiophene-based building blocks predicated by DFT decreased in the order of **DTT** > **TT** > **BTT** > **BT** > **T**. The optimized geometries and HOMO levels of the various thiophene derivatives were consistent with the evolutions of the UV–vis spectra. Among them, the **DTT** building block featured an entirely planar geometry, along with the strongest electron-donating ability (i.e., the most up-shifted HOMO level), which endowed **CP-DTT** with the strongest D-A interaction, most effective π-conjugation, and, thus, most red-shifted light absorption with the narrowest *E*_g_.

### 2.3. PHP of the **CPs**

**Figure 5:**
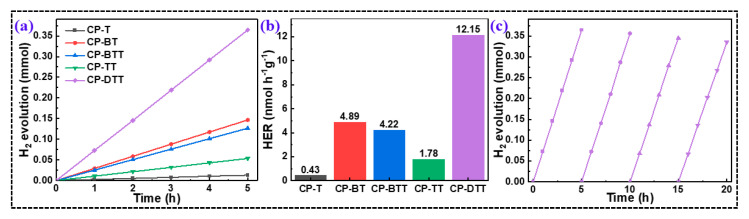
PHP as a function of time of 6 mg CPs dispersed in AA/H_2_O under visible light (**a**) and normalized HERs (**b**) based on a PHP cycle test of **CP-DTT** (**c**).

The above structure–property correlations and FMO levels of CPs indicate that all five CPs were suitable for the PHP reaction, with sufficient driving force for proton reduction. We tested the PHP performances (Figure 5) of the CPs by using ascorbic acid (AA) as a sacrificial electron donor (SED) under visible light irradiation (λ > 420 nm). All CPs could be well-dispersed in H_2_O without the aid of a water-soluble organic co-solvent to assist wettability. Furthermore, a co-catalyst was not added to provide redox reaction sites and reduce the activation energy of the reaction. The hydrogen production rates (HERs) of **CP-T**, **CP-BT**, **CP-BTT**, **CP-TT**, and **CP-DTT** were 0.43, 4.89, 4.22, 1.78, and 12.15 mmol h^−1^ g^−1^, respectively, following the order of **CP-DTT > CP-BT > CP-BTT > CP-TT > CP-T** (Figure 5b), which matched well with the trend of the PL intensity and TPR, as mentioned above. The HER of 12.15 mmol h^−1^ g^−1^ for **CP-DTT** was above the average of the abovementioned types of organic photocatalysts and outperformed most of the reported linear CPs (Table 1). Among all CPs, the single-thiophene-based **CP-T** showed the lowest HER (0.43 mmol h^−1^ g^−1^). The HERs of the twisted and unfused **BT**- and **BTT**-based CPs **CP-BT** and **CP-BTT** were nearly equal to each other (4.89 vs. 4.22 mmol h^−1^ g^−1^), meaning that the increase in the unfused thiophene numbers was not conducive to the enhancement of PHP performance, mainly because of the reduced intrinsic charge mobility. For the fused thiophene TT- and DTT-based CPs, the photocatalytic HERs were 1.78 and 12.15 mmol h^−1^ g^−1^, following the order of **CP-DTT** > **CP-TT** > **CP-T**, showing that increasing the number of fused thiophene in polymers is beneficial for PHP.

The stability of the best-performing **CP-DTT** was further checked by the recycling test. No significant reduction in continuous PHP was observed. After four cycles of **CP-DTT** for 20 h, about 92% of the initial H_2_ evolution (0.34 mmol) remained. This means that **CP-DTT** showed good stability for PHP (Figure 5c).

## 3. Materials and Methods

### 3.1. Materials and Methods

There was no further purification of all of the starting reagents. Anhydrous toluene was distilled freshly with calcium hydride (CaH_2_). The standard Schlenk techniques were used for all polymerizations.

The Bruker Advance III 400 model 400 MHz NMR spectrometer (Berlin, Germany) was used for NMR measurements (the sample was dissolved with 0.5 mL of deuterium reagent). All theoretical calculations were carried out on Gaussian 09W Packs using the semi-empirical method by PM6 [47,48,49]. The chemical structure was optimized and characterized at 298 K by frequency analysis. An FT-IR spectrometer (Bruker, ALPHA, Berlin, Germany) was used to obtain FT-IR spectra in the range of 4000–500 cm^−1^ (using the KBr compression method at a pressure of 1.0 T). The morphologies were determined by SEM (MLA650F, FEI, Hillsboro, OR, USA) (Working distance: 5 mm and magnification: 50–200,000 times). A UV-2600 scanning UV-vis spectrophotometer (Schimadzu, Kyoto, Japan) was used to characterize the DRS spectra (reference substance: BaSO_4_ and scanning range: 200–800 nm). A HORIBA Instruments FL-1000 fluorescence spectrometer (Kyoto, Japan) was used to study the PL spectra. The powdery **CP-T**, **CP-BT**, **CP-BTT**, **CP-TT**, and **CP-DTT** were excited by 437, 445, 460, 477, and 562 nm wavelengths, respectively. The normal three-electrode cell system was used for CV measurement, which was carried out on a CHI660E (Chenhua, Shanghai, China) electrochemical workstation. In the electrode system, platinum wire was the counter electrode, the Ag/AgCl electrode was the reference electrode, and the glassy carbon electrode was the working electrode. The supporting electrolyte was 5 mL acetonitrile used to dissolve tetra-n-butylammonium hexafluorophosphate (TBAPF6, 1.5 g). To characterize the TPR, an electrochemical workstation (CHI650E/700E, Shanghai, China) was used, which was equipped with a conventional three-electrode system. In the electrochemical workstation, the platinum plate was used as the counter electrode and the configuration Ag/AgCl (saturated with KCl) was used as the reference electrode.

### 3.2. Synthesis of Dibromocyanostilbene (DBCS)

DBCS was obtained through the classic Knoevenagel condensation reaction from simple starting chemicals. The specific steps are as follows: 4-bromophenylacetonitrile (389 mg, 2 mmol), 4-bromobenzaldehyde (406 mg, 2.2 mmol), and ethanol (0.7 mL) were added to a round-bottomed flask containing a solution of KOH (aq. 40%, 0.46 mL) and ethanol (0.46 mL) [36]. The reaction mixture was stirred at 30 °C for 1.5 h and the resulting solid was filtered and recrystallized from water/methanol (*v*:*v* = 20:1) to produce the compound as a white solid (700.6 mg, 96.5%). ^1^H-NMR (400 MHz, CDCl_3_) δ: 7.75 (d, *J* = 8.0 Hz, 2H), 7.59 (m, 4H), 7.53 (d, *J* = 12.0 Hz, 2H), 7.50 (d, *J* = 8.0 Hz, 2H), 7.45 (s, 1H), 7.40 (d, *J* = 12.0 Hz, 2H), 7.29 (s, 1H), 7.23 (d, *J* = 8.0 Hz, 1H), and 7.02 (d, *J* = 8.0 Hz, 2H); ^13^C-NMR (100 MHz, CDCl_3_) δ:143.3, 141.1, 133.1, 132.5, 132.3, 132.1, 131.1, 130.7, 130.4, 127.5, 124.6, 123.9, 114.0, 111.4, and 100.0.

### 3.3. Synthesis of **CPs**

**CP-T, CP-BT, CP-BTT, CP-TT**, and **CP-DTT** were prepared with DArP. **DBCS** (121 mg, 0.33 mmol) with **T** (28 mg, 1 equiv), **BT** (55.3 mg, 1 equiv), **BTT** (82.7 mg, 1 equiv), **TT** (46.7 mg, 1 equiv), **DTT** (65.3 mg, 1 equiv), Pd_2_(dba)_3_ (4.6 mg, 1.5 mol %), P(*o*-MeOPh)_3_ (3.5 mg, 3 mol %), anhydrous Cs_2_CO_3_ (434.4 mg, 2 equiv), PivOH (10.3 mg, 30 mol%), and toluene (5 mL) was added to Schlenk tubes [50,51,52,53]. Each reaction system was deoxidized by a reiterative vacuum and argon filling. To remove dissolved air, the freeze–vacuum–thaw cycle methods were performed for the mixture and it was then rigorously stirred at 130 °C for 48 h under an argon atmosphere. When the reaction mixture was cooled to room temperature, the solvent was removed with CH_2_Cl_2_ and the reaction residue was cleaned. The soluble impurities and inorganic salts in the undissolved crude CPs product were washed successively with CH_2_Cl_2_, ethanol, and water. Then, the product on the filter paper was moved to a vacuum for 24 h at 65 °C. Eventually, polymeric powders were obtained for **CP-T**, **CP-BT**, **CP-BTT**, **CP-TT**, and **CP-DTT** with yields of 19.7%, 88.9%, 83.6%, 66.1%, and 89.2%, respectively.

### 3.4. PHP Tests

For the typical PHP test, a gas chromatograph (GC9790, FuLi, Wenzhou, China) was equipped with a thermal conductive detector, using argon as the carrier gas. It was linked to a photocatalytic online analysis system (LabSolar-III AG, Beijing Perfect Light, Beijing, China). Five grams of AA were dissolved in 30 mL of H_2_O; the mixed aqueous solution was prepared for the ultrasonic dispersal of the photocatalysts (6 mg). The KOH solution was used to adjust the pH to 4.0. To remove the dissolved air from the mixture and maintain the vacuum, an oil pump was used. A 300 W Xe lamp (Beijing Perfect Light, PLS-SXE300, Beijing, China) under full-arc light irradiation was prepared to irradiate the reaction vessel. A flow of cooling water was used to keep the reaction temperature at 25 °C.

## 4. Conclusions

In summary, a series of polymer photocatalysts based on thiophene (**T**), unfused thiophene (**BT** and **BTT**), and fused thiophene (**TT** and **DTT**) were successfully designed and synthesized by atom-economic direct C–H arylation for visible-light-driven hydrogen evolution. Specifically, without adding any co-catalysts or co-solvents, **CP-DTT** showed the highest hydrogen evolution efficiency of 12.15 mmol h^−1^ g^−1^ (λ > 420 nm light irradiation). The results showed that the fused thiophene ring broadened light absorption, narrowed the band gap, enhanced the photocurrent intensity, and then improved the photocatalytic performance. The photocatalytic performance of the CPs increased with the increase in the amount of fused thiophene amount, but chain thiophene was not affected by the number. For the unfused dithiophene and terthiophene, the increase in the number of thiophene rings enabled more rotation freedom between the thiophene rings and thus decreased the intrinsic charge mobility of polymeric chains, resulting in lower hydrogen production performance. This work is the first systematic study to have been conducted on controlling the number of thiophene rings and its effect on polymer photocatalysts‘ properties and photocatalytic performance, which provides useful guidance for the design of thiophene-containing CP photocatalysts for PHP applications.

## Figures and Tables

**Table 1:** Summary of the PHP performances of several linear CPs.

Photocatalyst	Synthesis Methods	Co-Catalyst	SED	λ (nm) ^a^	HER (mmol h^−1^ g^−1^)	Ref.
**CP-DTT**	DArP	--	AA	>420	12.15	This work
P10	Suzuki	--	TEA	>420	3.26	[3]
CP4	DArP	--	AA/SA	>420	0.17	[29]
CP3	DArP	--	AA	>420	7.60	[32]
P3HT	Ni-catalyzed	Pt	Na_2_S/ Na_2_SO_3_	>400	0.005	[37]
PFBT/CN	Suzuki	Pt	TEOA	>420	0.72	[38]
P7-E	Sonogashira	--	TEOA	>420	6.02	[39]
L-PDBT-O	Sonogashira	--	TEOA	>420	4.43	[40]
Flu-SO	Suzuki	--	TEA	>420	5.04	[41]
FSO-FS	Suzuki	--	TEOA	>420	3.40	[42]
PyPm	Suzuki	Pt	TEOA	>300	0.37	[43]
p-FuS	Suzuki	--	TEA	>420	5.88	[44]
FSO-TPdT	Suzuki	Pt	TEOA	>420	7.39	[45]
PEB−DBT−0.1PY	Sonogashira	--	TEOA	>420	0.54	[46]

AA: ascorbic acid, SA: sodium ascorbate, TEOA: triethanolamine, and TEA: trimethylamine. ^a^ All light sources are a 300 W Xe lamp.

## Data Availability

Not applicable.

## Supplementary Materials

The following supporting information can be downloaded at: https://www.mdpi.com/article/10.3390/molecules28114507/s1. Figure S1. ^1^H NMR spectra of **DBCS** in CDCl_3_. Figure S2. ^13^C NMR spectra of **DBCS** in CDCl_3_. Figure S3. CV curves of the as-prepared CPs. Figure S4. Tauc plots of the transformed Kubelka–Munk function vs. energy of CPs.

## Sample Availability

Samples of the compound **CPs** are available from the authors.
