# Supplementary material for: Effect of Controlling Thiophene Rings on D-A Polymer Photocatalysts Accessed via Direct Arylation for Hydrogen Production
Source: Molecules. 2023 Jun 1;28(11):4507. doi: 10.3390/molecules28114507 (PMC10254606; doi:10.3390/molecules28114507)
Supplement: Supplementary file 1 [file molecules-28-04507-s001.zip › molecules-2395073-supplementary.pdf]

Supporting Information

For

Effect of Controlling Thiophene Rings on D-A Polymer Photocatalysts Accessed via Direct Arylation for Hydrogen Production

Dongnai Ye [1,2], Lei Liu [1], Qimin Peng [1], Jiabin Qiu [1,*], Hao Gong [1], Aiguo Zhong [3] and Shiyong Liu [1,*]

[1] Jiangxi Provincial Key Laboratory of Functional Molecular Materials Chemistry, College of Materials, Metallurgical and Chemistry, Jiangxi University of Science and Technology, Ganzhou 341000, China; yedongnai2014@gnnu.edu.cn (D.Y.); 13125257083@139.com (L.L.); qm2474572831@163.com (Q.P.); 6720210821@mail.jxust.edu.cn (H.G.)

[2] School of Chemistry and Chemical Engineering, Gannan Normal University, Ganzhou 341000, China

[3] Department of Pharmacy & Chemistry, Taizhou University, Taizhou 318000, China; zhongaiguo@tzc.edu.cn

* Correspondence: jiabinqiu@jxust.edu.cn (J.Q.); chelsy@jxust.edu.cn or chelsy@zju.edu.cn (S.L.)

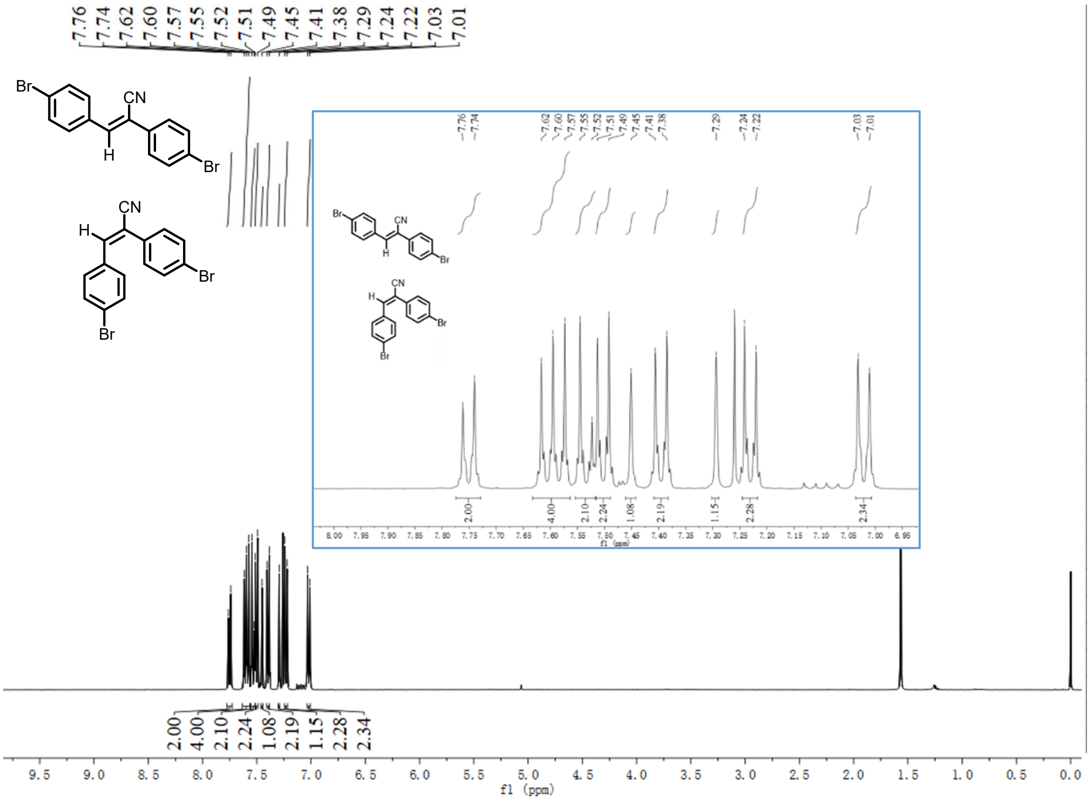

Figure. S1. ^{1}H NMR spectra of **DBCS** in $CDCl_3$.

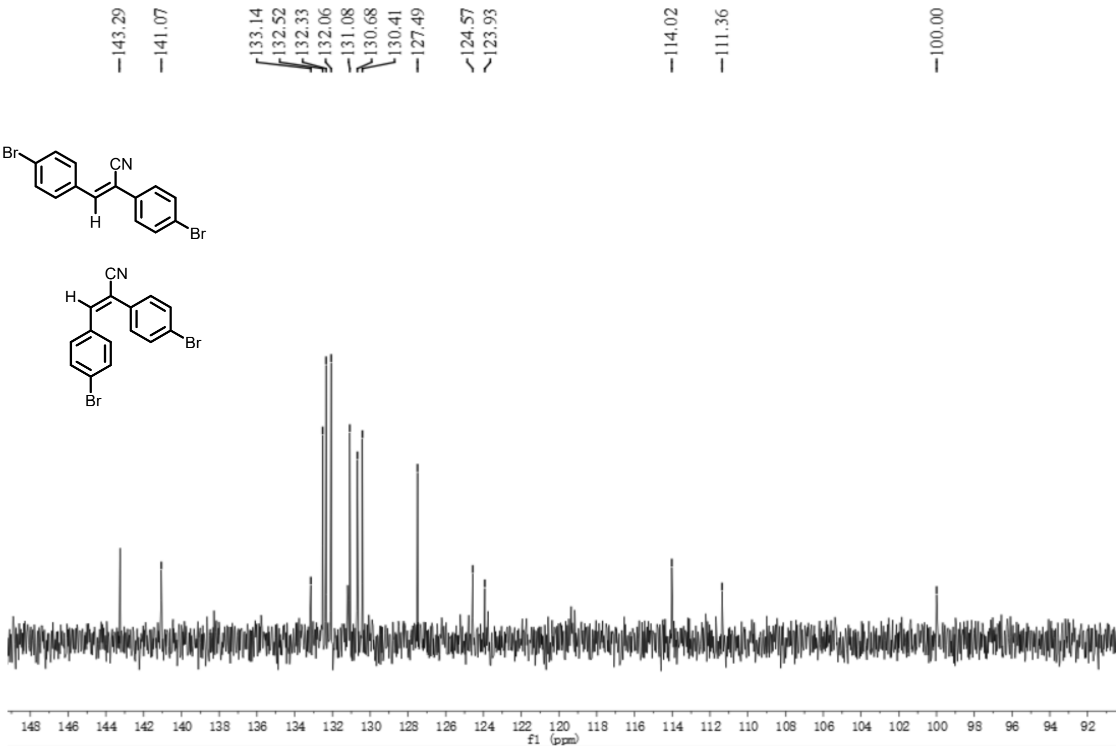

Figure. S2. ^{13}C NMR spectra of **DBCS** in $CDCl_3$.

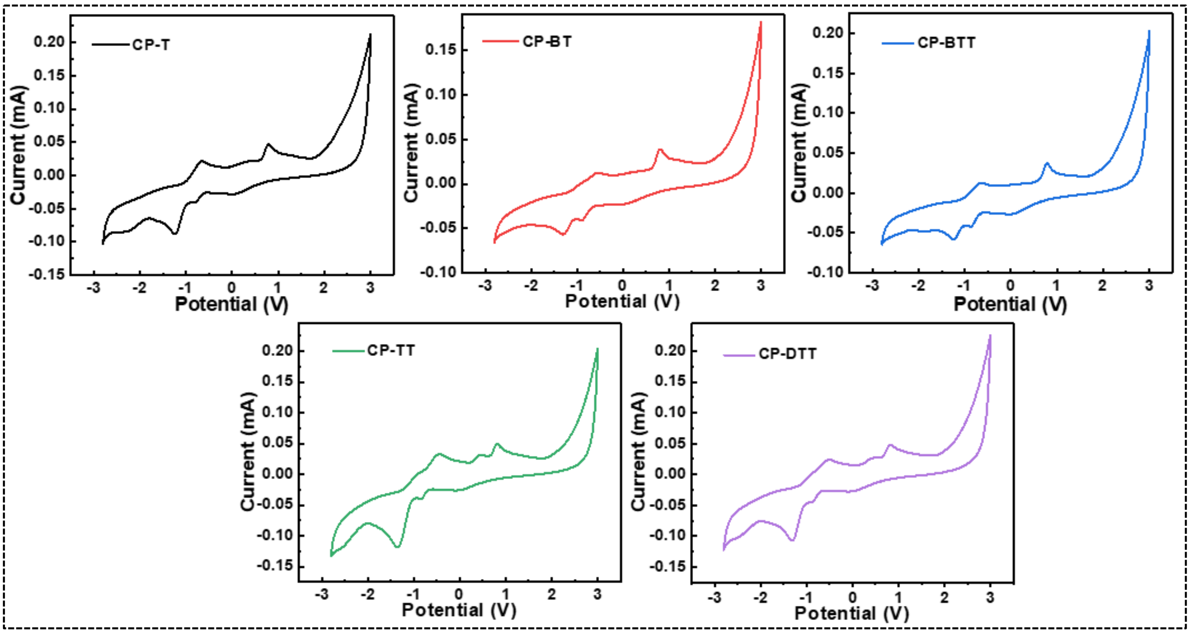

Figure. S3. CV curves of the as-prepared CPs.

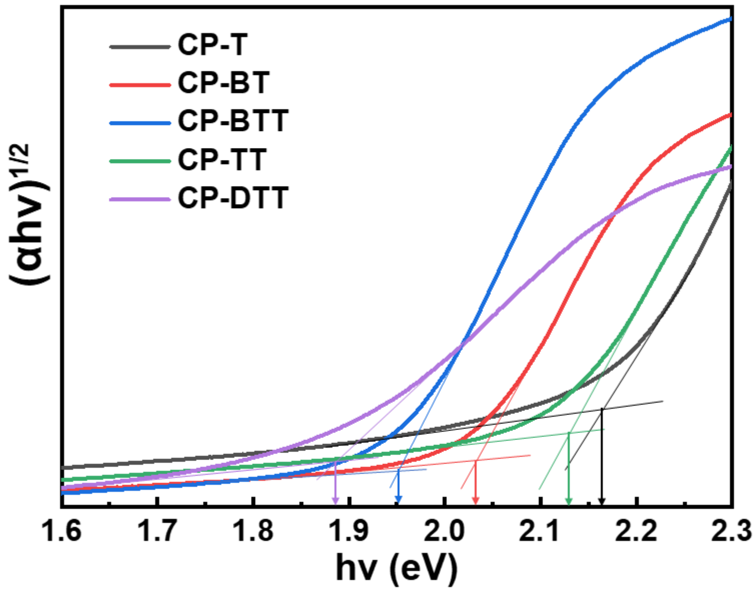

Figure. S4. Tauc plots of the transformed Kubelka−Munk function vs energy of CPs.
